# Programmable multi-DNA release from multilayered polyelectrolytes using gigahertz nano-electromechanical resonator

**DOI:** 10.1186/s12951-019-0518-7

**Published:** 2019-08-06

**Authors:** Xinyi Guo, Hongxiang Zhang, Yanyan Wang, Wei Pang, Xuexin Duan

**Affiliations:** 1 State Key Laboratory of Precision Measuring Technology & Instruments, Tianjin University, Tianjin, 300072 China; 2 College of Precision Instrument and Opto-electronics Engineering, Tianjin University, Tianjin, 300072 China

**Keywords:** Controllable release, NEMS resonator, Gigahertz ultrasound, Acoustic streaming, Micro-vortexes, Polyelectrolyte thin films

## Abstract

**Background:**

Controllable and multiple DNA release is critical in modern gene-based therapies. Current approaches require complex assistant molecules for combined release. To overcome the restrictions on the materials and environment, a novel and versatile DNA release method using a nano-electromechanical (NEMS) hypersonic resonator of gigahertz (GHz) frequency is developed.

**Results:**

The micro-vortexes excited by ultra-high frequency acoustic wave can generate tunable shear stress at solid–liquid interface, thereby disrupting molecular interactions in immobilized multilayered polyelectrolyte thin films and releasing embedded DNA strands in a controlled fashion. Both finite element model analysis and experiment results verify the feasibility of this method. The release rate and released amount are confirmed to be well tuned. Owing to the different forces generated at different depth of the films, release of two types of DNA molecules with different velocities is achieved, which further explores its application in combined gene therapy.

**Conclusions:**

Our research confirmed that this novel platform based on a nano-electromechanical hypersonic resonator works well for controllable single and multi-DNA release. In addition, the unique features of this resonator such as miniaturization and batch manufacturing open its possibility to be developed into a high-throughput, implantable and site targeting DNA release and delivery system.

**Electronic supplementary material:**

The online version of this article (10.1186/s12951-019-0518-7) contains supplementary material, which is available to authorized users.

## Background

Controlled release of drugs, especially macromolecular therapeutic agents such as DNA, is commonly adopted in a variety of fields, spanning from the basic researches of biomedical materials to the application development of gene-based therapies [1–3] due to their precise control of the dosage, minimum side-effect and high treatment efficacy [4]. To realize an effective controllable release, numerous immobilization and encapsulation approaches have been applied for the establishment of drug carriers [5–8], among which one of the most extensively used and most promising methods goes to the self-assembly of polyelectrolytes through layer-by-layer (LbL) technique [9, 10]. The precise and nanometer-scaled control over film thickness and drug capacity of this method has been highlighted by numerous researchers [11, 12]. Besides, simply by adopting a certain condition that can induce film disruption, release of DNA and other biological molecules can be achieved. So far, approaches to promoting LbL film disruption have been studied extensively [13–15], including (a) methods based on environment changes, such as pH [16, 17], ionic strength [18] and liquid temperature [19, 20], (b) methods using specific materials that participate in certain kinds of chemical reactions, such as reductively [21], enzymatically [22, 23] and hydrolytically [24] degradable polyelectrolytes, and (c) methods by applying external stimulus, such as light [25], electrochemical potentials [26, 27] and ultrasound [28, 29]. Each of these outlined approaches keeps their respective strengths, and many have been verified to be suitable for controlled DNA release [30–33]. However, their disruptions rely either on critical environment factors or special chemical property of the polyelectrolytes, which bring additional restrictions for practical use [11]. For example, intense environmental changes required in certain release process may put forward a higher request for the protection of molecular bioactivity and restrict the application of the method in cell experiments and living organisms, and the adoption of some special materials in some cases to enhance the disruption may increase the complexity and cost of DNA immobilization.

Another focus in the LbL-based release is the programmable release of multiple biological agents [34–37]. Controlling the release rate of several targets in different orders, such as sequential or parallel release, or even separate and mutually exclusive release profiles, can provide effective tools for combined drug therapy investigation and achieve a better efficacy. Till now, several studies have achieved the multiple release of different DNA constructs assembled by polyelectrolyte films, and most of them adopts specific design of complex film materials and structures to achieve the required release behavior [32, 38–40]. For example, Liu et al. [38] demonstrated a film fabrication method using a set of specially designed degradable cationic polymers performing different erosion speeds, which was applied to release two different plasmids with distinct profiles; Jessel et al. [39] reported the use of cationic cyclodextrins as an enhancer for sequential and direct delivery of different DNA molecules into cells in contact with the films. Therefore, developing DNA release method that can realize controllable and multiple release with simple and moderate disruption condition is in great demand.

Owning to the development of microsystem and nanotechnology, acoustic devices based on piezoelectric materials have gained increasing attention in biochemical research field [41–44] which is due to their low cost, batch manufacturing, small volume and noninvasive to biomolecules [45–47]. Here, we demonstrated a novel and versatile controlled release approach using gigahertz ultrasound (hypersound) induced by a nano-electromechanical acoustic resonator composed of ultra-thin material layers (several tens to hundreds of nanometers thick). The ultra-short attenuation distance of this high frequency ultrasound wave provides a steep acoustic gradient at concentrated active region, thus generates micro vortexes which can offer a powerful shear stress on the interfaces between the vortexes and the substrates and effectively realize DNA release from polyelectrolyte films deposited on surface. Results of our experiments verifies that by tuning the power applied to the device and the distance between device and LbL films, DNA release rate and amount can be precisely controlled. We also designed a multi-DNA release system by simply assembling two kinds of DNA molecules with commonly used cationic polymers into LbL films. The porous film property making possible for flowing liquid to pass through the nano-sized pores and interact with DNA molecules seated on inner layers. Thus, concurrent release with distinct properties of two kinds of DNA molecules which are located on different depth of the films was achieved due to the gradually decreased fluid velocity and shear stress from the outer layer to the inner layer. Other advantages of this method such as mild and pure physical interactions, simple operation and low power consumption (several hundreds of milliwatts) open possibilities for it to be further developed into a universal, high-throughput and implantable in vivo DNA release and delivery system.

## Methods

### Materials

Poly (allylamine hydrochloride) (PAH, MW = 120,000–200,000) and linear poly (ethylene imine) (LPEI, MW = 25,000) were obtained from Alfa Aesar (United States). Poly (sodium 4-styrenesulfonate) (PSS, MW = 70,000) was purchased from Sigma Aldrich Co. (United States). Single-stranded DNA molecules of 75 base pairs (5′ to 3′: (T)_15_CTAACTGC TGGGCGATTCTGGTGACGCGGCAACGATGATTGGGAACGATGATTGGGAACA) were synthesized by Sangon Biotech Co., Ltd. (Shanghai, China), and were labeled by Alexa Fluor 488 (DNA-Green) or CY3 (DNA-Red). All chemicals were used as received without any further purification. Deionized water (DI water, 18.25 MΩ) was used for the preparation of buffer, polymer and DNA solutions.

### Preparation of the LbL films

Prior to film preparation, glass substrates and QCM chips were cleaned by 5 min rinsing in ethanol, 5 min rinsing in DI water, nitrogen-blow drying, and 20 min oxygen plasma treatment. 2 mg/ml PAH, 2 μM DNA, 28 mg/ml PSS and 10 mg/ml LPEI used for the fabrication of multilayered films were prepared in the presence of 150 mM NaCl (pH = 6.5). Solution of LPEI contains 10 mM HCl to facilitate polymer solubility. 6 bilayers of PAH/DNA-Red were achieved using a layer-by-layer (LbL) method: substrates were alternatively submerged in PAH and DNA-Red solutions (15 min each), and were rinsed in a 150 mM NaCl solution (pH = 6.5) for 5 min between the deposition of each two layers. Multilayered films composed of [LPEI/PSS]_3_/PAH/DNA-Green/[PAH/PSS]_5_/PAH/DNA-Red/PAH were applied in multiple DNA release experiments, and its buildup procedures were operated as described above. In QCM experiments, films were assembled under a flowing condition (100 μl/min) assisted by a peristaltic pump (Ismatec, ISM596D) and a flow module (Q-Sense, QFM 401), and the incubation time of each layer lay on QCM frequency variation rate.

### Device fabrication

Fabrication process of a nano-electromechanical hypersonic resonator is illustrated in Additional file 1: Figure S1. Briefly, alternating layers of silicon dioxide (SiO_2_) and aluminum nitride (AlN) were deposited on Si wafer using plasma-enhanced chemical vapor deposition (PECVD) and reactive sputtering respectively to form the Bragg mirror for acoustic reflection. A 600 nm thick molybdenum (Mo) layer was further deposited by RF sputtering and patterned by plasma etching to form the bottom electrode. After that, 1000 nm AlN was deposited as the piezoelectric layer and patterned by reactive ion etching (RIE) to expose bottom electrode for electrical connection. Finally, 60 nm chromium (Cr) and 300 nm gold (Au) were evaporated and patterned by lift-off process, serving as top electrodes and testing pads.

### Controlled release system

Sinusoidal signal (1.56 GHz) applied to the resonator was generated by a signal generator (Agilent, N5171B) and pre-amplified by a power amplifier (Mini-Circuits, ZHL-5W-422+), and resonators were wire-bonded to evaluation boards (EVB boards) for signal transmission and device performance characterization. Polydimethylsiloxane (PDMS) chambers with different heights were sealed on resonator substrates to form liquid containers. During DNA release experiments, PDMS chambers were fulfilled with 150 mM NaCl solution (pH = 6.5), and glass substrates modified with multilayered films were covered on the top of the chamber. By controlling power output of the generator, hypersonic wave and micro-vortexes in liquid can be stimulated and well-tuned. In QCM measurements, 200 μl NaCl solution was added to an open module (Q-Sense, QOM 401) loaded with a modified QCM chip. A T-shaped EVB board with a resonator facing down was designed and inserted into the solution during DNA release process, and the distance between the resonator and QCM chip was controlled to 200 μm.

### Characterization

Fluorescence microscope (Olympus BX53) with a CCD camera (Olympus DP73) was utilized to examine the amount of DNA-Red remained on glass substrates during LbL and DNA release process. Before characterization, glass slides were rinsed with DI water and dried in nitrogen. Fluorescent pictures of a fixed area on each slide were taken at each time point using an exposure time of 100 ms, and the fluorescence intensity was calculated using ImageJ. Fluorescent value in liquid was detected using a Microplate spectrophotometer (Thermo Scientific, VARIOSKAN LUX, 548 nm/580 nm). Quartz crystal microbalance (QCM, Q-Sense) was used to provide mass information, and all measurements were performed at 35 MHz resonant frequency. Surface topography of multilayered films was characterized by scanning electron microscope (SEM, FEI F50), and 2 nm Au was evaporated on glass substrates before SEM characterization to enhance surface conductivity. Film thickness was recorded by atomic force microscope (Bruker, Dimension Icon, tapping mode). In multiple DNA release experiments, DNA-Red and DNA-Green were measured by a fiber optic spectrometer (NOVA-EX, Ideaoptics, China). A scan range from 500 to 700 nm was used, and different optical filters were applied to ensure that only one peak will be recorded in one measurement. The fluorescent intensities of these two molecules were extracted at 580 nm and 537 nm respectively. Gel electrophoresis was used to characterize the integrity of DNA molecules after the treatment of acoustic streaming. DNA samples (prepared in 150 mM NaCl solution) were loaded into 1.0 wt% agarose and ran at 120 V for approximately 30 min, and then the gel was photographed under UV light using a fluorescent gel imaging and analysis system (ChemiDoc XRS+, BIO-RAD).

## Results and discussion

### Release system and mechanism study

**Fig. 1:**
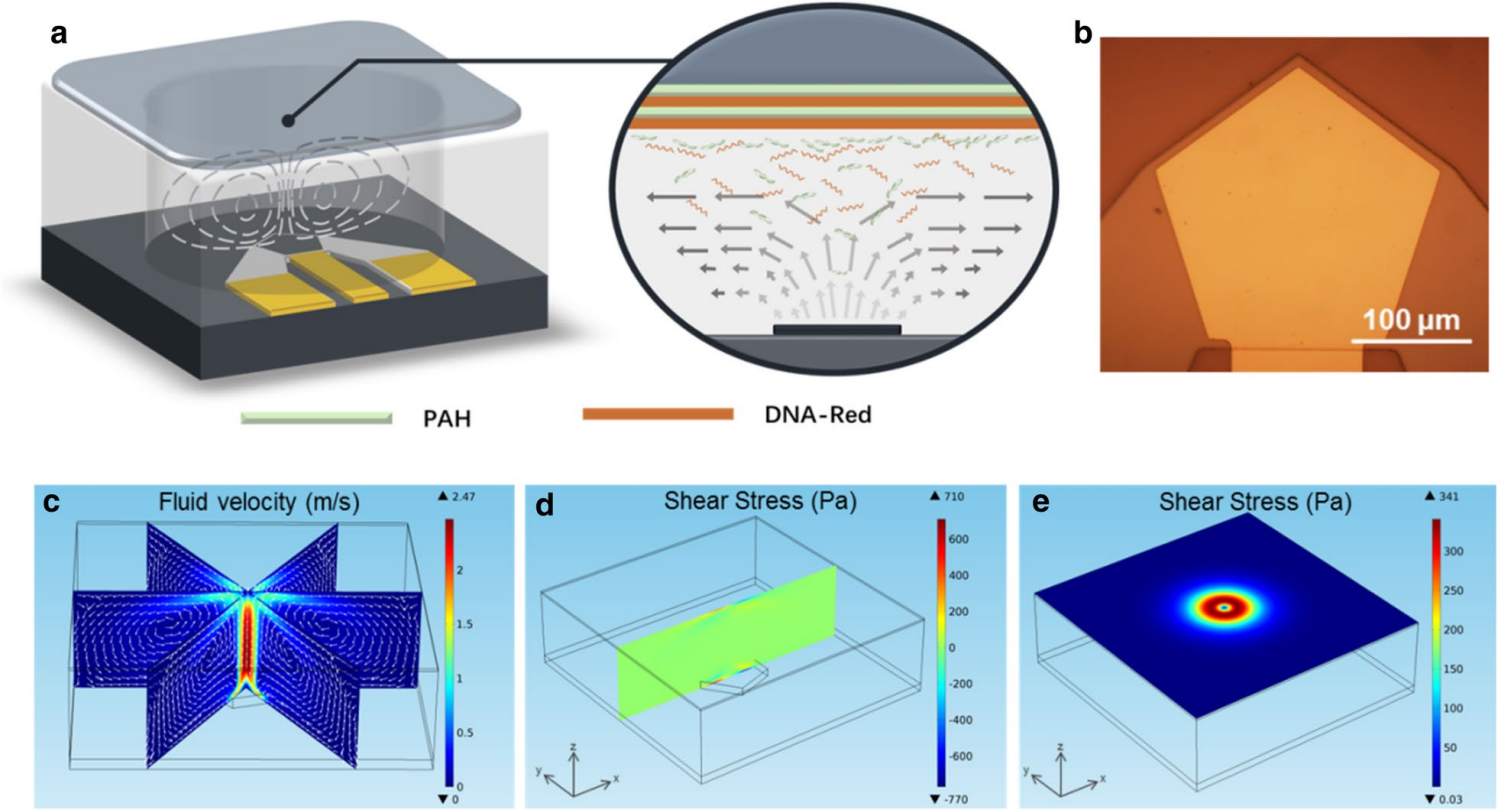
Release system based on nano-electromechanical resonator and theoretical simulation. **a** Schematic of release system. Multilayered films composed of [PAH/DNA]_6_ were deposited on glass surface through LbL technique. Micro-vortexes triggered by the nano-electromechanical hypersonic resonator generate controlled shear forces on adsorbed molecules, disassemble the multilayered films, and thus release embedded DNA strands. **b** Top view of the resonator under microscope. **c**–**e** 3D FEM analysis of acoustic streaming and resulted shear force. **c** Micro-vortexes formed under the stimulation of a hypersonic resonator at 1.56 GHz. Color bar indicates the distribution of fluid velocity and arrows describe flow direction. **d** Shear stress distribution along x-direction on the plane 200 μm away from resonator surface. **e** Composited shear stress distribution on x–y plane 200 μm away from resonator surface

To realize the delicately controlled DNA release, a delivery system integrated of nano-electromechanical (NEMS) hypersonic resonator was established and shown in Fig. 1a. The NEMS resonator is composed of a piezoelectric layer sandwiched between two metal electrodes which is fabricated by CMOS compatible process. Optical image of the device is shown in Fig. 1b, and electrical property of the device is characterized which shows a resonant frequency at 1.56 GHz (Additional file 1: Figure S2). The golden pentagon on device surface indicates top electrode and working area of the device, and the thickness of each material layer in a resonator ranges from several tens to several hundreds of nanometers. To form a stable and adjustable test condition, PDMS chambers with a diameter of 6.9 mm were mounted and sealed on the resonator. 150 mM NaCl solution was filled in the chamber during release experiments to imitate an environment adaptable to human body fluid. Glass slides modified with target molecules using LbL technique were covered on the chamber and in contact with the liquid.

Our previous works have confirmed that under resonator stimulation, the propagation and attenuation of acoustic waves in liquid will form stable and powerful micro-vortexes [48]. Generally, the energy leakage of sound waves causes its pressure to decrease during its propagation in liquid, and this part of assumed power is converted to the momentum of flow motion. The amplitude (A) of an acoustic wave can be described as

1 $${\text{A}} = A_{0} *e^{ - \beta z}$$

where $A_{0}$ is the initial wave amplitude, $\beta$ is the attenuation coefficient, and z is the distance between acoustic source and measured point. The coefficient $\beta$ describes the attenuation rate, and is given by

2 $$\upbeta = \frac{{2\upmu\omega^{2} }}{{3\rho c^{3} }}$$

Here, $\upmu$ indicates fluid viscosity, $\omega$ denotes acoustic frequency, $\rho$ is liquid density and $c$ is the sound velocity in liquid. The equation clearly shows that acoustic attenuation is frequency-squared dependent, and a higher frequency leads to a much stronger energy decrease. For example, when travel 200 μm in fluid, sound waves of 1.5 GHz have already dissipated to 3% of its initial amplitude, but waves of 150 MHz still have 96% remained. Hence, traditional ultrasound (kHz–MHz) can hardly generate powerful acoustic streaming, and controllable release methods based on ultrasound usually rely on cavitation [28]. In our hypersonic resonator system, the elevated acoustic frequency (1.56 GHz) can provide a higher fluid velocity, which certifies it to be a preferable tool for the generation of localized high-speed micro-vortexes which can be more moderate and easier controlled. The three-dimensional finite element model (3D FEM) analysis of the acoustic streaming is shown in Fig. 1c. Liquid above the pentagonal working area of the resonator is accelerated by device resonation, moves upward from the center, and returns through the edge. When the uplifted fluid reaches the interface between liquid and solid, which is glass substrates modified with DNA molecules in our work, flow direction is forced to change. Fluid disperses laterally, and the longitudinal component of fluid velocity is attenuated to zero in a short distance. The vertical (z direction) gradient of the lateral fluid velocity ($V_{x}$) causes shear stress ($\uptau$) at the border, which can be defined by the formula

3 $$\uptau =\upmu\frac{{\partial V_{x} }}{\partial z}$$

This shear stress interacts with materials at the interface, overcomes the electrostatic forces among polyelectrolytes, and finally leads to the disassembly of the multi-layered films including the embedded chemicals. Figure 1d shows the one-dimensional distribution of shear stress along x-direction on the top border 200 μm away from the resonator surface, and a two-dimensional composited stress is described in Fig. 1e. Most intense shear stress can be seen focused right above the fringe of device working area, and gradually decreases outwards. Although the value of shear stress outside the pentagon region seems to be much smaller from the simulation, our experimental results indicate that it’s enough for multilayer film disassembly, and the release efficiency is not restricted by the small size of the hypersonic resonator.

### Film disassembly triggered by a nano-electromechanical hypersonic resonator

In this work, in order to attest to the universality of this method, commercialized and commonly used polycation PAH was used, and multi-layered films simply composed of six bilayers of PAH/DNA-Red (single-stranded DNA labeled by CY3) were applied as a release model. To confirm that the quality of our established film is adequate for the following release experiments, deposition process was monitored by fluorescence microscope (Additional file 1: Figure S3), and the steady increase of fluorescent intensity indicates the successful built up of uniform multi-layered polyelectrolyte thin films.

**Fig. 2:**
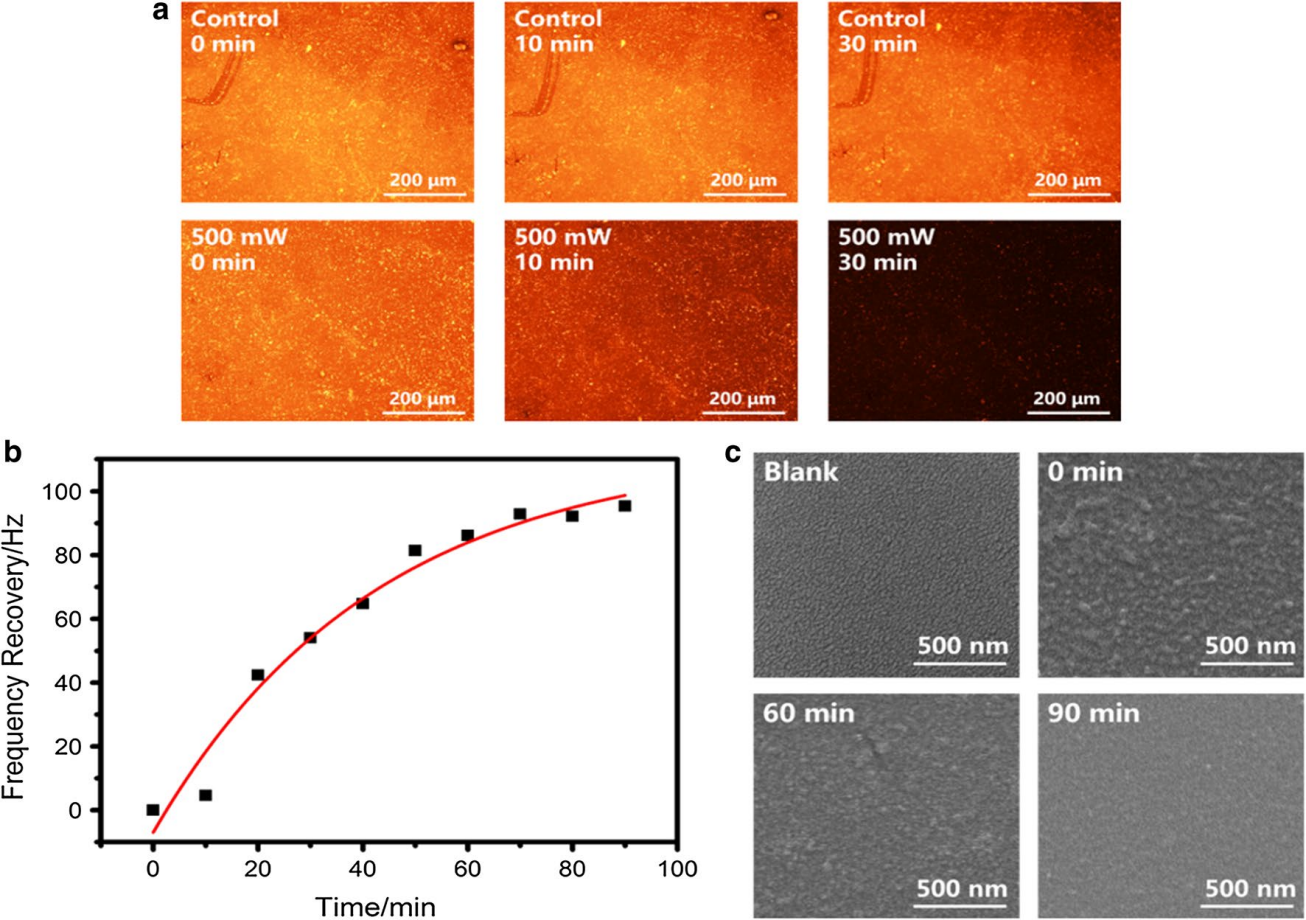
Characterization of DNA release process. **a** Fluorescent images of LbL films immersed in buffer without (control group) and with (experiment group, 500 mW) resonator stimuli, observed at 0, 10 and 30 min. **b** QCM frequency recovery during DNA release. Release effectiveness was observed from baseline changing each time after 10 min treatment. The exponential fit has a correlation coefficient of 0.973. **c** SEM images of bare glass (blank) and LbL films before (0 min) and after (60 min and 90 min) resonator stimuli

To trigger the film destruction and DNA release, signal of 500 mW power at 1.56 GHz was applied to the resonator, and the remaining fluorescent DNA on glass substrate was recorded (Fig. 2a). No obvious differences can be observed from the control group (without resonator stimuli) during 30 min incubation, indicating a good stability of DNA-Red embedded in polyelectrolytes. Meanwhile, fluorescent intensities of the experiment group present a sharp decrease. This clear distinction of the release behavior proofs that the hypersonic resonator can effectively disassemble the electrostatically adsorbed DNA molecules. To confirm that this phenomenon is not caused by the disruption of fluorescent molecules, we further detected the variation of fluorescent intensities in solution (Additional file 1: Figure S4). The growth of fluorescent value in liquid over treating time is consistent with the result observed from glass surface, and the quantitative analysis indicates a final DNA concentration of 9.3 nM.

To further prove the film release, mass and morphology change of LbL films were also recorded in our study. Real-time mass monitoring of the release process was carried out using QCM (Additional file 1: Figures S5, S6; Fig. 2b). The mass change follows an exponential decay, and 90 min are required to stop the frequency recovery. Film morphology was further characterized by SEM (Fig. 2c). Glass substrate presents a smooth surface with fine texture before film deposition. After LbL modification, a much rougher structure with dense nano-sized islands and particles was obtained. After 90 min treatment, hardly any sediment remains, and the glass substrate almost returns to its original state. AFM detection of film thickness (Additional file 1: Figure S7) also reveals that only 20.5% of the initial thickness remains after 90 min treatment. All these results indicate the efficient removal of the materials adsorbed on glass surface. Besides, the structural integrity of DNA molecules with or without resonator stimuli was analyzed by agarose gel electrophoresis (Additional file 1: Figure S8). All lanes migrated to the same position, proving that this approach can realize DNA release without producing any appreciable structural damages.

### Controlled DNA release

According to the fluorescent and QCM measurement, we conclude that film disassembly and DNA release induced by the NEMS resonator is a progressive rather than an immediate process. Therefore, this technology is very suitable for sustained drug release in a controlled manner. Here, to further study the release kinetics and realize controlled DNA release, two variables, power applied to the resonator (hereinafter, power) and distance between resonator and LbL films (hereinafter, height), are chosen to be optimized. (Other factors influencing DNA release are discussed in Additional file 1: Figures S9 and S10).

**Fig. 3:**
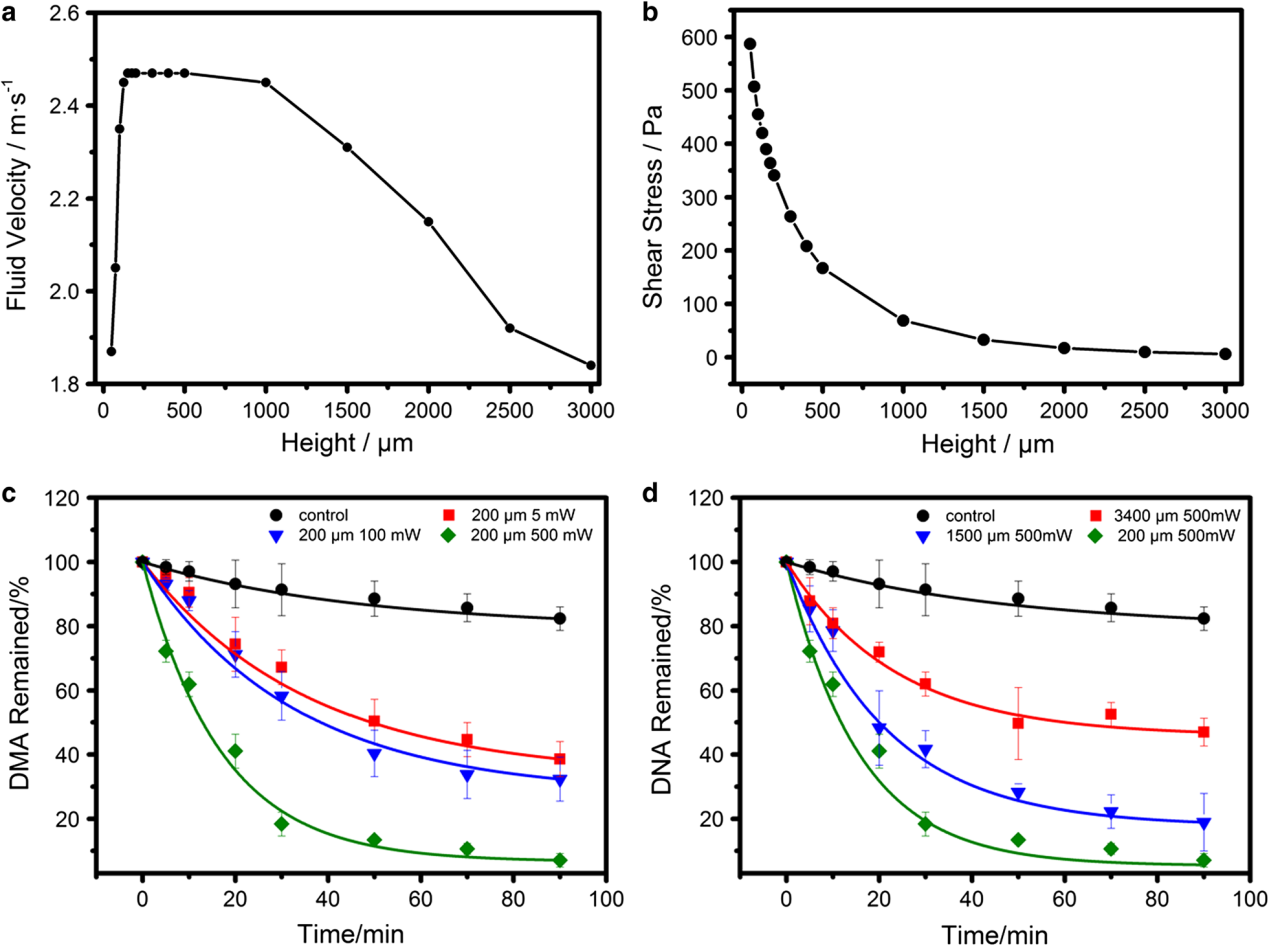
Tunable DNA release. Variation trends of maximum vortex speed (**a**) and interface shear stress (**b**) when changing the height were theoretically analyzed using FEM simulation. Experimental release effectiveness under different power (**a**) and height (**b**) were recorded using percentage of remained DNA as a function of resonator treating time, observed at 0, 5, 10, 20, 30, 50, 70 and 90 min

Theoretically, power determines the energy absorbed by liquid, thus influences the speed of the vortexes. With higher power applied, vortexes can reach a higher velocity, attain a larger shear force within the same distance, and thus realize a faster release rate. On the other hand, under same power condition, height change produces much more complex effects on the vortex formation (Fig. 3a). When the height is too small, the accelerating distance of liquid above device surface is limited, thus restricts the generation of high-speed vortex. When it is too large, the large liquid volume actuated by a single device will also constrain the maximum fluid speed. However, the shear stress at solid–liquid interface shown in Fig. 3b continues to decay exponentially within the entire height range. This is due to the change of distance between the vortex velocity center (where the maximum velocity locates) and the interface. The maximum velocity in the vortex occurs about 60 μm away from resonator. When the height is small, liquid–solid interface locates very close to the velocity center, thus the resulted shear stress can be large. On the contrary, a larger height will lead to more severe energy attenuation during the upward movement of fluid, and the actual influence of fluid on interface will be weakened, thereby further reduce its ability for film disassembly. In summary, by altering power and height, shear stress changes monotonically, and different release rates can also be achieved.

To confirm our deduction, experiments with three different powers (5 mW, 100 mW and 500 mW) and heights (200 μm, 1500 μm, and 3400 μm) were carried out, and the results demonstrate a power and height dependent release character consistent with the above analysis (Fig. 3). Curve fitting in the figure shows an exponential trend, and we can express this regularity by the following equation

4 $${\text{y}} = Y_{0} + A *e^{{ - R_{0} t}}$$

**Fig. 4:**
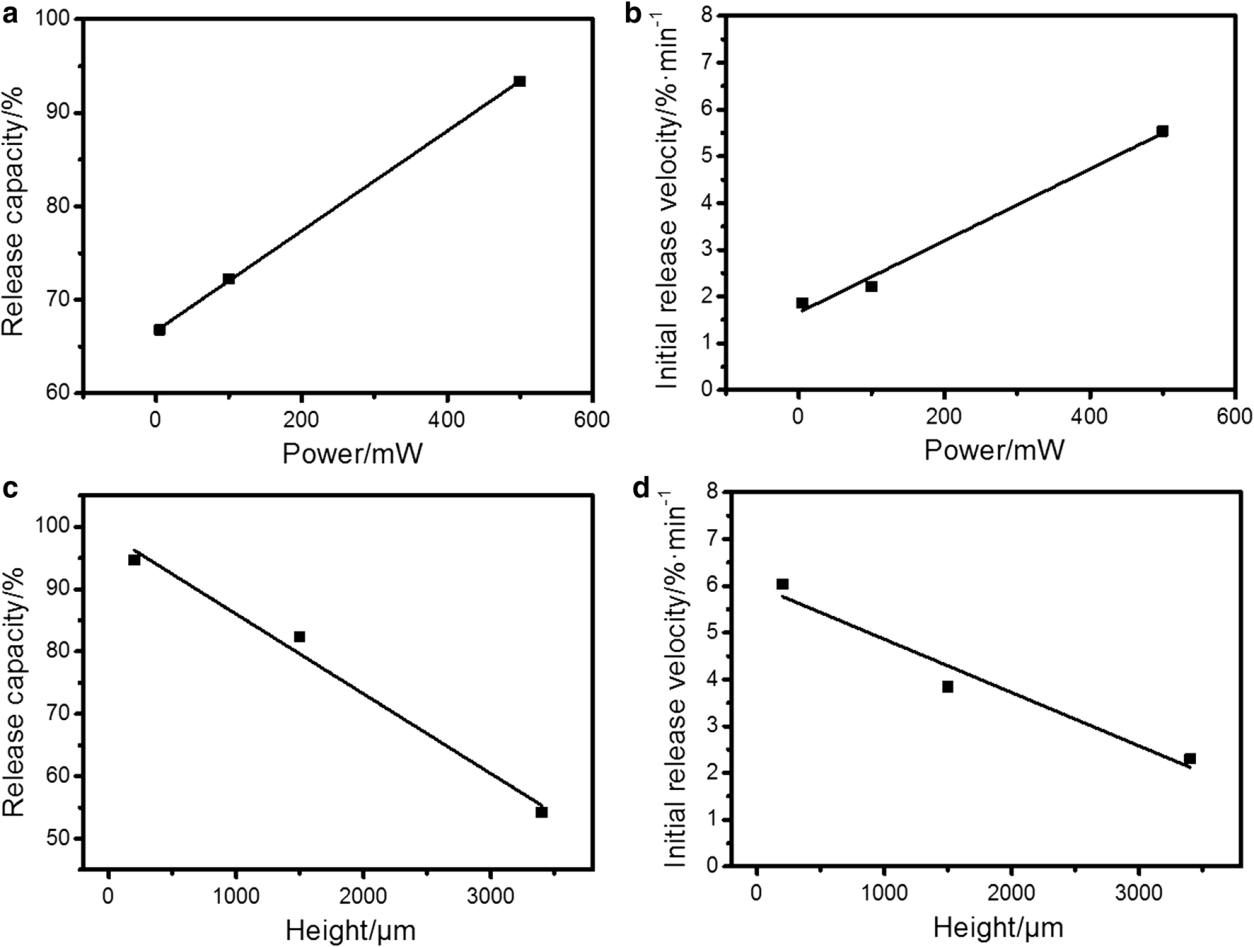
Analysis of DNA release rate and amount. Release capacity (100%-$Y_{0}$, indicating theoretical DNA amount which can be released from surface) and initial release velocity ($A *R_{0}$) were extracted from fitting curves in Fig. 3c, d and plotted here as a function of power (**a**, **b**) and height (**c**, **d**)

Here, $Y_{0}$, $A$ and $R_{0}$ are three parameters determined by power and height. When treating time (t) is sufficient enough, y can be represented by ${\text{Y}}_{0} ,$ which indicates ultimate DNA amount remained on surface. $- A *R_{0}$ refers to curve slope when $t = 0$, and can be used to describe the initial release rate. Thus, quantitative analysis of release rate and released amount can be achieved by calculating $Y_{0}$ and $A *R_{0}$. Their specific values were extracted from fitted curves and were plotted in Fig. 4 as a function of power and height. We can conclude from the figures that by altering height and power, the release velocity and ultimate released quantity vary linearly. This character provides us an opportunity to preset power and height from a calibration curve according to required release effectiveness.

### Multiple DNA release and mechanism study

The results above have demonstrated that a hypersonic resonator can realize a well-controlled release of a single kind of DNA molecule. To meet the requirements of multiple DNA release, two kinds of DNA strands were embedded in multilayered films in our following studies, and the release character and mechanism were thoroughly studied.

**Fig. 5:**
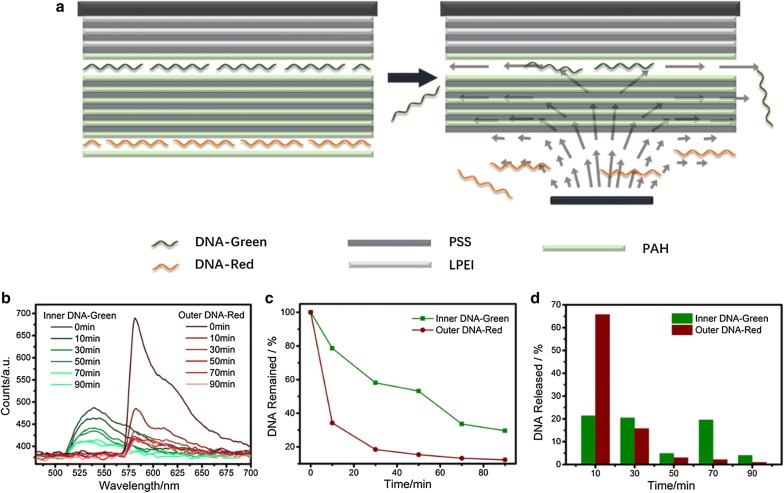
Characterization of multi-DNA release. **a** Schematic of film composition and release process for multiple DNA release. **b** Spectrum result of DNA remained on glass surface under resonator stimuli, observed at 0, 10, 30, 50, 70 and 90 min. Fluorescent intensities of DNA-Green and DNA-Red were calculated at 537 nm and 580 nm, respectively. Power and height were set to 500 mW and 200 μm. **c** Percentage of remained DNA as a function of resonator treating time. **d** Differential percentage of DNA released during each period of observation

Figure 5a describes the modification procedure used in this section. DNA-Red (labeled by red fluorescent molecule, CY3) and DNA-Green (labeled by green fluorescent molecule, AF488) were sequentially embedded at different depth of the films. These two molecules are identical except the fluorescent marker to guarantee that the release effectiveness will not be influenced by their structures and properties. Substrates were pre-coated with 3 LPEI/PSS bilayers to improve interfacial property and DNA adsorption. 5 bilayers of PAH/PSS were inserted between two kinds of DNA molecules for better distinction. Fluorescent spectrums of these two DNA molecules were separately recorded using a fiber optic spectrometer system, and the results are displayed in Fig. 5b. Figure 5c shows the remained DNA percentages extracted at 580 nm and 537 nm, and differential analysis of DNA released during each period of observation is given in Fig. 5d.

**Fig. 6:**
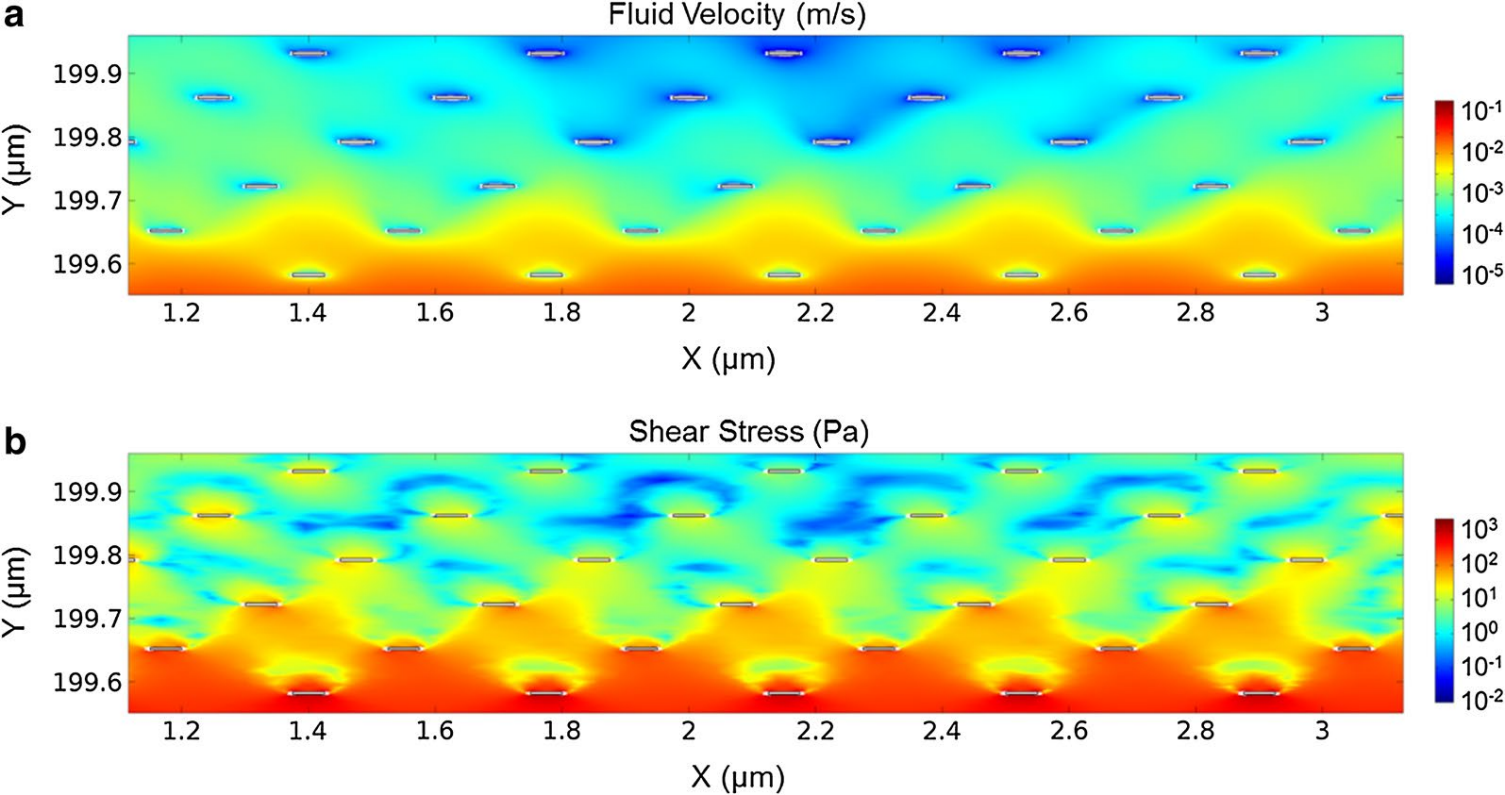
Simulated distributions of **a** fluid velocity and **b** shear stress within LbL films. White stripes indicate cross-sections of polymer and DNA molecules, and are staggered and dispersed in colored fluid area to represent the interlaced and porous film structure

A clear result obtained from Fig. 5 is that instead of a sequential and outside-in release character, deep-seated DNA-Green and shallow-seated DNA-Red are released concurrently, except that the release rate of inner DNA-Green appears to be slower. This phenomenon is attributed to the structure of polyelectrolyte films established by LbL technique, which has been reported to exhibit a porous and permeable property [49]. During the release process, the up-flowing liquid stands a chance to pass through the nano-sized pores and reach the innermost films, thus creates shear force on inner surfaces and further release part of loosely bound DNA molecules. The inhibition of outer materials makes the force descend with the increase of depth, and a smaller shear force exerted on inner molecules will evidently lead to a lower release rate. To verify our analysis, 2D-FEM simulation is given in Fig. 6, in which staggered white stripes are applied to represent molecule cross-sections for model simplification, and other colored area indicates the space among molecule strings. The intervals are amplified to provide a clear observation of field distribution. The hypersonic resonator is placed at the lower right corner of the simulation area (not shown here), and the distance between the resonator and films is set at 200 μm. Under resonator stimuli, gradually varied color can be seen in the graphic, indicating gradually decreased fluid velocity and shear stress from the outer layer to the inner layer, thus confirms our explanations.

Here, a detailed description can be provided for the entire multiple DNA release process. When hypersound treatment started, both DNA-Red and DNA-Green release according to the fluid velocity and shear stress they received. After 10 min, over 60% of outer DNA-Red has been removed. Only small amounts of DNA-Red who bond tightly to or even embedded in the internal materials are left on the surface, which lead to a much lower release rate. After 30 min, the release of inner DNA-Green also begins to slow down, indicating that most of loosely bound DNA-Green molecules have been successfully released. 20 min later, the release of outer DNA-Red is almost accomplished, and its velocity approaches to zero. Meanwhile, a slightly increased speed was observed on inner DNA-Green (Fig. 5d), which can be explained by the larger shear force obtained due to the entire removal of exterior materials and the exposure of DNA-Green to the vortexes.

These results clearly indicate that our NEMS resonator is able to realize a differentiable multi-DNA release. Different release rates can be obtained simply by using different embedding levels. There are no requirements in the selection of polycation electrolytes and the structural difference between the target DNA molecules. Since the device is CMOS compatible, the applied power can be actually programmed, a tunable release of two or even more kinds of target DNA molecules can be achieved, which holds great significance in the area of medical applications.

## Conclusions

In summary, we developed a novel method to trigger the disassembly of multilayered polyelectrolyte thin films for controllable single and multi-DNA release using nano-electromechanical hypersonic resonators. Due to the ultra-short attenuation distance of hypersonic waves in liquid, localized and high-speed micro-vortexes are triggered, thus creating controlled shear forces at liquid–solid interface for film disruption. Simply by tuning the power applied to the device and the distance between device and embedded films, release speed and amount can be precisely controlled, demonstrating a good controllability of this approach. In addition, a unique feature that the vortexes can penetrate the multilayered films and create shear forces on inner layers, enables this approach to release multiple DNA molecules at the same time with different speed, providing a simple method without using complex assistant molecules for combined gene therapy. It is also noted that since the hydrodynamic approach is a pure physical method and the NEMS resonator is CMOS compatible, it can be readily applied to different types of controlled release applications.

## Additional file

**Additional file 1.** Additional figures and experimental details. **Figure S1.** Fabrication process of a nano-electromechanical hypersonic resonator. **Figure S2.** Electrical property of the resonator. **Figure S3.** Fluorescent observation of film assembly. **Figure S4.** Fluorescence of released DNA in liquid. **Figure S5.** Setup for QCM detection of film disassembly. **Figure S6.** Real-time results of QCM detection of film disassembly. **Figure S7.** Film thickness detection. **Figure S8.** DNA agarose gel electrophoresis. **Figure S9.** Temperature control. **Figure S10.** Influence of different temperature.

## Data Availability

All data supporting this study are included in this published article and its additional file.

## Abbreviations

LbL: layer-by-layer
PAH: poly (allylamine hydrochloride)
LPEI: linear poly (ethylene imine)
PSS: poly (sodium 4-styrenesulfonate)
DNA-Red: DNA molecules labeled by CY3
DNA-Green: DNA molecules labeled by Alexa Fluor 488
QCM: quartz crystal microbalance
DI water: deionized water
SiO_2_: silicon dioxide
AlN: aluminum nitride
Mo: molybdenum
Cr: chromium
PECVD: plasma-enhanced chemical vapor deposition
RIE: reactive ion etching
EVB: evaluation boards
PDMS: polydimethylsiloxane
SEM: scanning electron microscope
3D FEM: three-dimensional finite element model
