# Supplementary material for: Programmable multi-DNA release from multilayered polyelectrolytes using gigahertz nano-electromechanical resonator
Source: J Nanobiotechnology. 2019 Aug 6;17:86. doi: 10.1186/s12951-019-0518-7 (PMC6683436; doi:10.1186/s12951-019-0518-7)
Supplement: Supplementary file 1 — Additional file 1. Additional figures and experimental details. Figure S1. Fabrication process of a nano-electromechanical hypersonic resonator. Figure S2. Electrical property of the resonator. Figure S3. Fluorescent observation of film assembly. Figure S4. Fluorescence of released DNA in liquid. Figure S5. Setup for QCM detection of film disassembly. Figure S6. Real-time results of QCM detection of film disassembly. Figure S7. Film thickness detection. Figure S8. DNA agarose gel electrophoresis. Figure S9. Temperature control. Figure S10. Influence of different temperature. [file 12951_2019_518_MOESM1_ESM.docx]

**Additional file 1**

Programmable Multi-DNA Release from Multilayered Polyelectrolytes Using Gigahertz Nano-Electromechanical Resonator

Xinyi Guo ^1^, Hongxiang Zhang ^2^, Yanyan Wang ^1^, Wei Pang ^2^, and Xuexin Duan ^1^*

^1^ State Key Laboratory of Precision Measuring Technology & Instruments, Tianjin University, Tianjin 300072, China

^2^ College of Precision Instrument and Opto-electronics Engineering, Tianjin University, Tianjin 300072, China

* Correspondence: [xduan@tju.edu.cn](mailto:xduan@tju.edu.cn)

**S1. Fabrication process of a nano-electromechanical hypersonic resonator**

A nano-electromechanical resonator is fabricated as shown in Figure S1. 3 bilayers of SiO_2_-AlN are first deposited for acoustic reflection. Mo, AlN and Au are then deposited and patterned to form the bottom electrode, piezoelectric layer and top electrode.


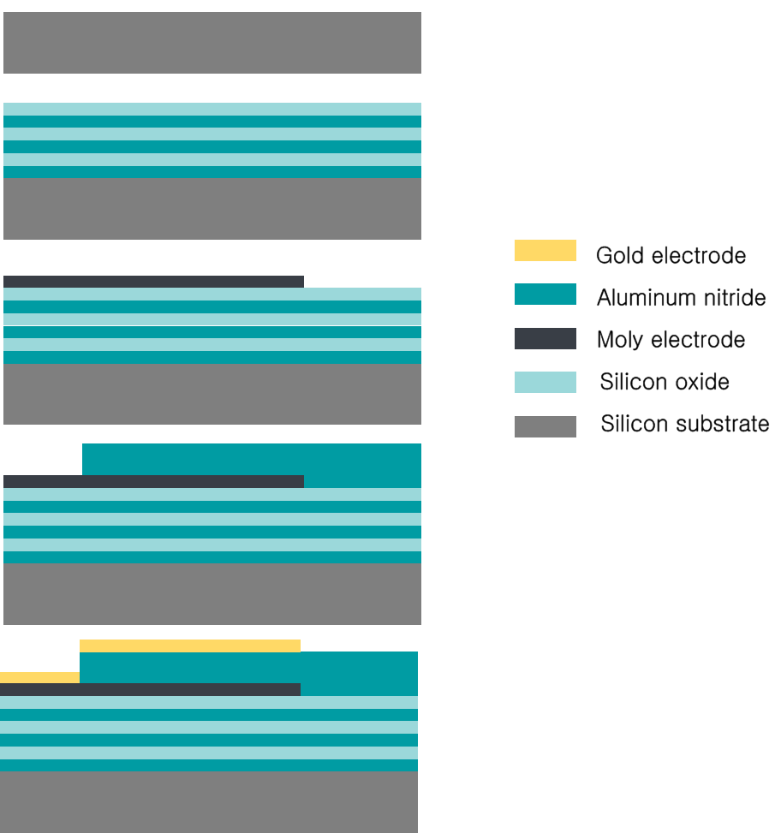


**Figure S1**. Fabrication process of a nano-electromechanical resonator.

**S2.** **Electrical property of the resonator**

Electrical property of the resonator was tested every time before an experiment to ensure the stability of device performance. The displayed Smith chart certifies our resonator to have a resonant frequency at 1.56 GHz and a satisfactory performance with a quality factor over 200.


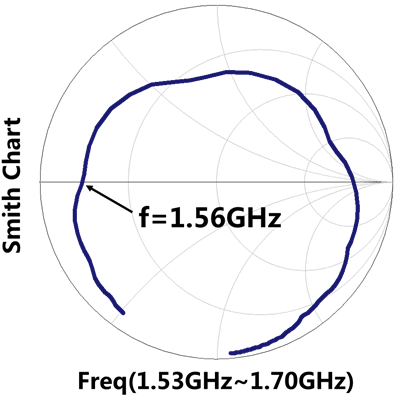


**Figure S2.** Smith chart of the resonator, which shows a good electrical property with a resonant frequency of 1.56 GHz.

**S3.** **Fluorescent observation of film assembly**

Fluorescent images were taken during the adsorption process, as shown in Figure S3 (a). The calculated fluorescent intensity is given in Figure S3 (b), which indicating the successful built up of multi-layered films.


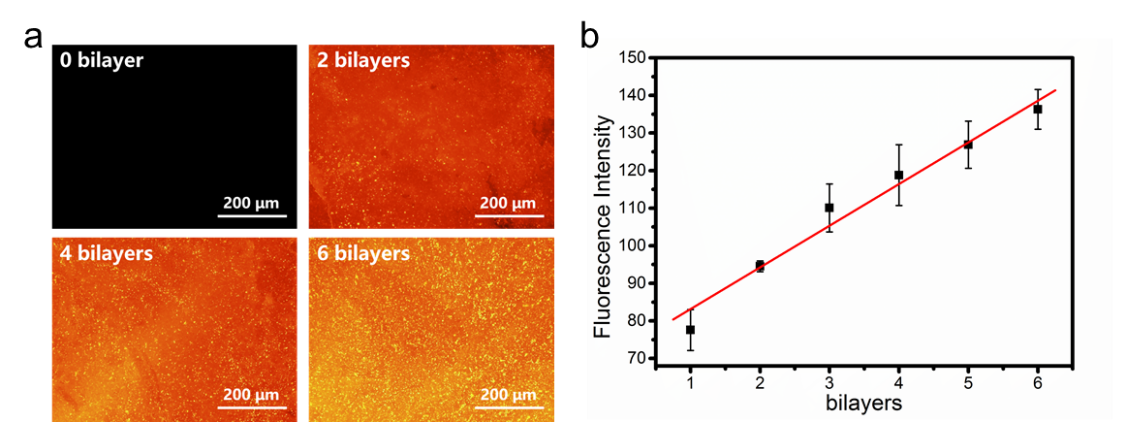


**Figure S3.** (a) Fluorescent pictures before (bare glass, 0 bilayers) and after depositing 2, 4, 6 bilayers of PAH/DNA. (b) Increasing fluorescent intensity as a function of bilayer number.

**S4.** **Fluorescence of released DNA in liquid**

Fluorescent intensities in liquid were detected each time after treatment, and the value is converted to DNA concentration, as shown in Figure S4. The increase of fluorescent value in liquid is consistent with the result observed from glass surface, and the final concentration of released DNA can reach 9.3 nM.





**Figure S4.** Fluorescent intensities in liquid and calculated DNA concentrations.

**S5.** **Setup for QCM detection of film disassembly**

QCM real-time mass monitoring of the release process was carried out using setup shown in Figure S5. To establish a release system compatible with QCM open cell, a T-shaped EVB board was designed, and the nano-electromechanical hypersonic resonator was attached to its bottom. During experiment, the device was inserted into the solution facing the upper surface of QCM chip. The height of the resonator was controlled by a precise positioning system, and the distance between the resonator and the QCM chip with DNA modification was set to 200 μm.


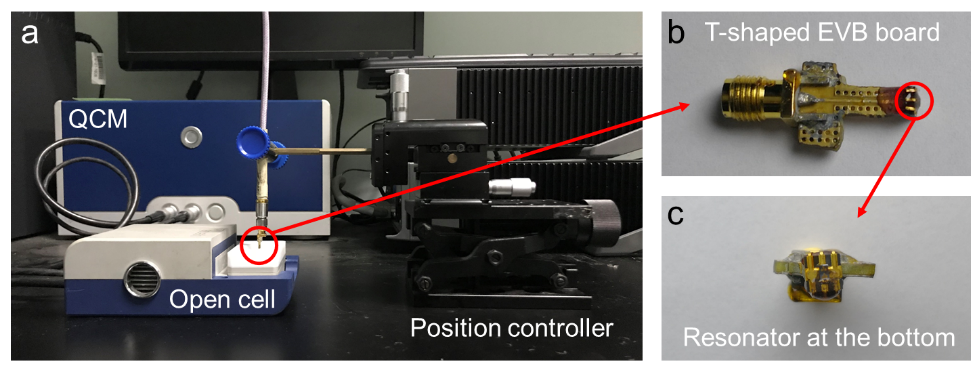


**Figure S5.** (a) QCM detection setup for the measurement of LbL disassembly. (b) T-shaped EVB board with (c) a resonator facing down was designed and inserted into the solution.

**S6.** **Real-time results of QCM detection** **of film disassembly**

Realtime QCM measurement of molecules remained on chip during resonator stimuli is given in Figure S6. Release effectiveness was observed from baseline changing each time after 10 min treatment. The sharp rise of frequency indicates resonator induced acoustic streaming.


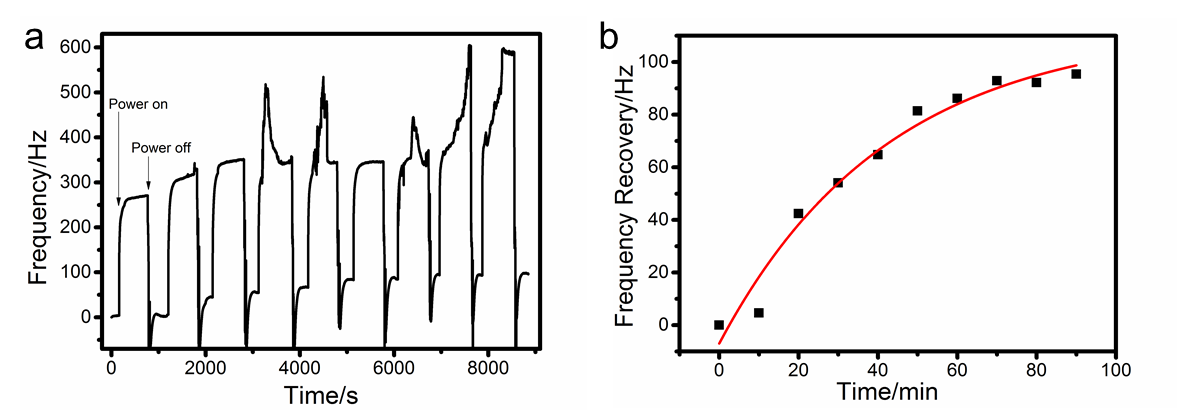


**Figure S6.** Realtime QCM measurement during resonator stimuli.

**S7. Film thickness detection.**

LbL film thickness before and after resonator treatment was detected by AFM, as shown in Figure S7. The average thicknesses are 34 nm (before treatment) and 7 nm (after treatment).


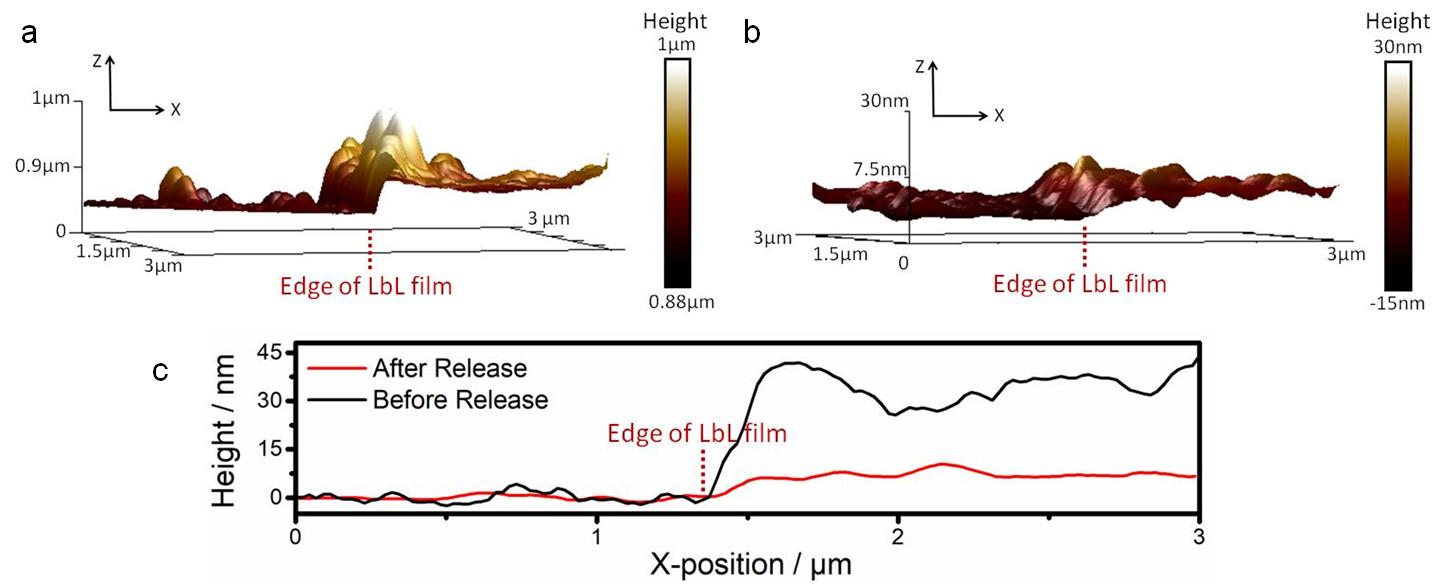


**Figure S7.** Film morphology recorded at film edge (a) before and (b) after 90 min resonator treatment. (c) Film thickness along X-direction.

**S8.** **DNA agarose gel electrophoresis.**

Structural integrity of DNA molecules with or without resonator stimuli was analyzed by agarose gel electrophoresis, as shown in Figure S8. No obvious differences between control and experimental group can be seen. The adjoining two lanes in each group may come from different DNA spatial structures existed in saline solution.


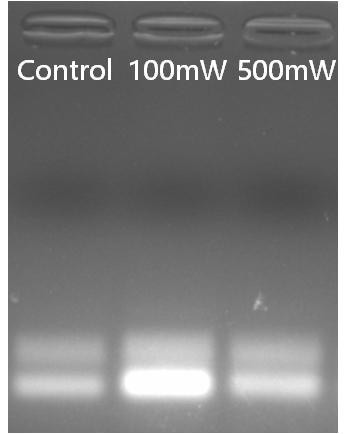


**Figure S8.** Agarose gel electrophoresis of DNA solution before (control) and after resonator stimuli (90min treatment at 100 mW and 500 mW).

**S9.** **Temperature control**

Heating effect of the resonator under high power should be considered as one factor influencing DNA release behavior. When using a small power (for example, lower than 300 mW), temperature in the release chamber basically maintains room temperature (approximately 28 ℃). Once the applied power exceeds 500 mW, limited energy conversion efficiency of device will increase the temperature to over 50 ℃. Although a higher temperature can serve as a tool for release enhancement, not all drug molecules remains their biological activity in such environment. Thus, all experiments introduced in the article were carried out with a temperature control setup, and the final temperature under 500 mW can be restricted to below 38 ℃ (Figure S9), which is similar to human body temperature and do no harm to almost all kinds of biological molecules.


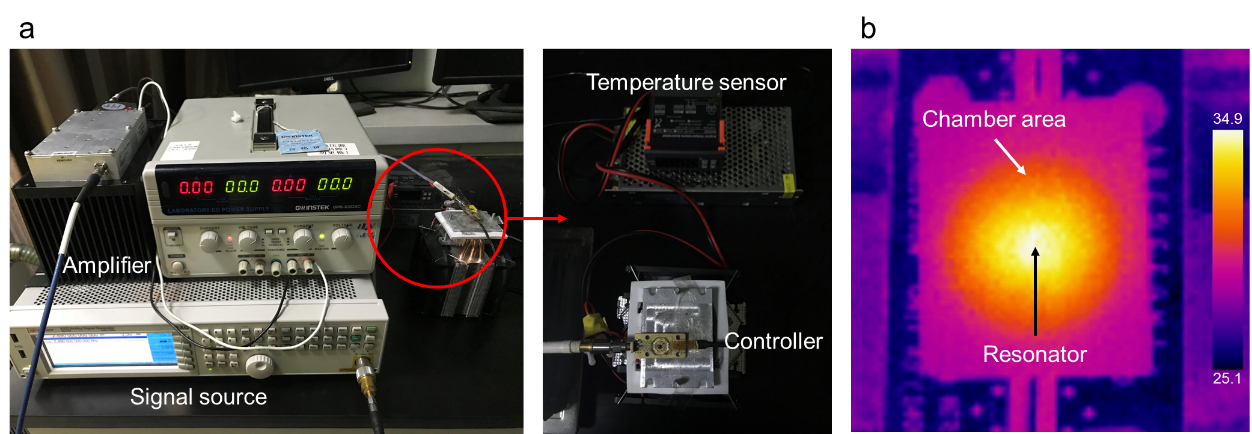


**Figure S9.** (a) Experiment setup with temperature control system. (b) The highest temperature in our experiments under 500 mW can be controlled to lower than 38 ℃.

**S10.** **Influence of different temperature**

Comparison of release rates in water bath (with no resonator stimuli) under 28 ℃ (room temperature) and 38 ℃ (final temperature in 500mW experiments assisted by temperature controller) provides a further confirmation that our release principle is hypersonic vortex induced shear force instead of temperature increase. This result also proves that our release system has the ability to be developed into a skin-contact or implantable drug release system without affecting normal human physical function.


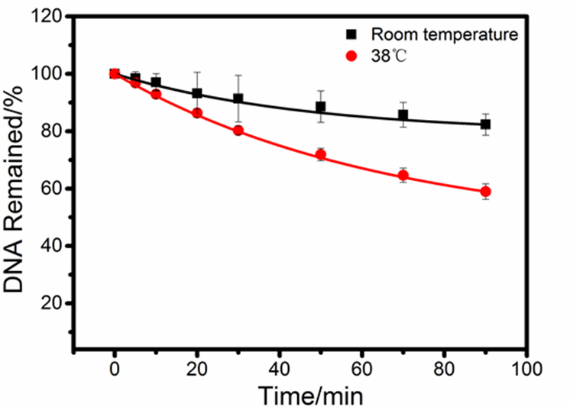


**Figure S10.** DNA remained on the surface without resonator stimuli under room temperature (black) and 38 ℃ water bath (red).
